# TRIM31 Deficiency Is Associated with Impaired Glucose Metabolism and Disrupted Gut Microbiota in Mice

**DOI:** 10.3389/fphys.2018.00024

**Published:** 2018-02-15

**Authors:** Jing Cheng, Fei Xue, Meng Zhang, Cheng Cheng, Lei Qiao, Jing Ma, Wenhai Sui, Xingli Xu, Chengjiang Gao, Panpan Hao, Meng Zhang, Yun Zhang

**Affiliations:** ^1^The Key Laboratory of Cardiovascular Remodeling and Function Research, Chinese Ministry of Education, Chinese Ministry of Health, The State and Shandong Province Joint Key Laboratory of Translational Cardiovascular Medicine, Shandong University Qilu Hospital, Jinan, China; ^2^Department of Immunology, Shandong University School of Medicine, Jinan, China

**Keywords:** TRIM31, glucose metabolism, insulin resistance, gut microbiota, inflammation

## Abstract

Tripartite motif-containing protein 31 (TRIM31), an E3 ubiquitin ligase of the tripartite motif family, plays an important role in the innate immune response. It can reduce the activity of the nucleotide-binding oligomerization domain-like receptor (NLR) family pyrin domain containing 3 (NLRP3) inflammasome. However, little information is about glucose metabolic health of TRIM31-deficient mice, and investigations about gut microbiota in TRIM31-deficient mice is limited. Thus, we aimed to compare glucose metabolic parameters, gut microbiota composition and inflammatory cytokine levels between TRIM31^−/−^ and wild-type (WT) mice, and further investigate whether or not certain gut microbiota taxon correlates with specific metabolic parameters and inflammation cytokines in TRIM31-deficient mice. TRIM31^−/−^ mice showed glucose intolerance and insulin resistance, with a significant difference in gut microbiota composition, characterized by increased abundance of Prevotellaceae and Veillonellaceae. TRIM31^−/−^ mice with impaired glucose metabolism was accompanied by elevated serum tumor necrosis factor-α (TNF-α) and interleukin 1β (IL-1β) concentrations, as well as upregulated caecal TNF-α, IL-1β, caspase-1, and NLRP3 expressions. Furthermore, elevated p-IRS-1/IRS-1 protein expression, and decreased Akt Thr308 phosphorylation were observed in TRIM31^−/−^ mice. Prevotellaceae abundance was positively associated with caecal IL-1β mRNA expression, and Veillonellaceae was associated with higher TNF-α mRNA expression and serum insulin concentration. In conclusion, our study is novel in showing that TRIM31 deficiency is associated with impaired glucose metabolism and disrupted gut microbiota in mice. This study contributes to the theoretical foundation on the potential relationship between TRIM31 deficiency and the development of abnormal glucose metabolism.

## Introduction

The prevalence of obesity and type 2 diabetes mellitus (T2DM) is increasing dramatically. However, the underlying mechanisms of the development of T2DM are still unclear. Insulin resistance and T2DM are strongly influenced by both genetic and environment factors (Hossain et al., 2007; Doria et al., 2008). It showed that low-grade chronic inflammationplayed an important role in the pathogenesis of insulin resistance and T2DM (Wellen and Hotamisligil, 2005). Recently, it indicated that environmental factors and host genetics can interact to control gut microbiota composition, which can contribute to the development of insulin resistanc and T2DM by triggering the immune response (Macdonald and Monteleone, 2005).

Gut microbiota can be regulated by innate immune system and the overall balance in gut microbiota composition is an important factor ensuring normal host functions. Gut microbiota dysbiosis can contribute to an expanding list of chronic and metabolic diseases (Spiller and Sloan, 2016). Tripartite motif (TRIM) family proteins are implicated in the negative regulation of innate immune responses (Versteeg et al., 2014). We recently found that TRIM31, an E3 ubiquitin ligase of the TRIM family proteins, may directly bind to the nucleotide-binding oligomerization domain-like receptor (NLR) family pyrin domain-containing 3 (NLRP3) and negatively regulate NLRP3 inflammasome activity (Song et al., 2016). NLRP3 inflammasome is a multi-protein platform comprising NLRP3, ASC, and caspase-1, and has a fundamental role in host defense against microbial pathogens (Guo et al., 2015). Subsequent evidence suggests that NLRP3 inflammasome activation can increase the susceptibility of several diseases, including obesity, insulin resistance, T2DM, and some autoimmune disorders (Wen et al., 2012).

Giving that TRIM31 plays a central role in regulating NLRP3 inflammasome activity and NLRP3 inflammasome activation can increase the risks of metabolic diseases, we aimed to determine glucose metabolic health, gut microbiota composition, and inflammatory cytokine levels in TRIM31^−/−^ mice, and further investigate whether or not certain gut microbiota taxon correlates with specific metabolic parameters and inflammation cytokines in TRIM31^−/−^ mice.

## Materials and methods

### Study approval

The study was approved by the Ethics Committee and the Scientific Investigation Board of Shandong University Qilu Hospital (Jinan, Shandong Province, China). All experimental procedures were performed in accordance with the recommendations found in the Guide for the Care and Use of Laboratory Animals published by the US National Institutes of Health (NIH publication no. 85–23 revised 1996).

### Mice and study design

Ten TRIM31^−/−^ mice on a C57BL/6J background were generated by Cyagen Biosciences Company. (Guangzhou, China) with transcription activator-like effector nuclease (TALEN) technology, as we previously described (Song et al., 2016; Liu et al., 2017). The genotyping of TRIM31^−/−^ mice was confirmed by sequencing PCR fragments (250 bp) in the TALEN-targeting region amplified with genomic DNA, isolated from mouse tail tips with the following primers: forward 5′-GGCCTTGGATTTCTGTACTTTCACATC-3′ and reverse 5′-TGGGCCTGAACGTATTCTTATTCACAG-3′. Age- and weight-matched male C57BL/6J wild-type (WT) mice were as controls (*n* = 10). All the mice had the same origin and were raised in the same condition. Experimental mice were genotyped by genomic DNA sequencing. The sequence analysis of WT and TRIM31^−/−^ mice involved the sequences CATTGACTGTGGGCACAACTTCTGCCTG and CATTGACTGTGGG-ACAACTTCTGCCTG (-1). The sequence peaks were shown in Figure S1. The mice were bred in the same room with 12/12-h light-dark cycles at the Animal Facility of Shandong University Qilu Hospital (Jinan, China). Mice were fed *ad libitum* with normal chow food and sterile water throughout the experimental period. In our study, only male mice were used to prevent potential confounding factors with the hormone profile of female mice.

### Glucose tolerance test and insulin tolerance test

We performed introperitoneal glucose tolerance test (IPGTT) and insulin tolerance test (ITT) in both 16- and 20-week old TRIM31^−/−^ and WT mice. Animals were given glucose (2 mg dextrose/g body weight) or insulin (1 U/kg body weight) by intraperitoneal injection, respectively. Then, blood glucose was measured before the injection (time 0) and at 15-, 30-, 60-, and 120-min intervals after injection. Blood glucose responses to the IPGTT and ITT was calculated as the area under the receiver operating characteristic curve (AUC) for each mouse according to the trapezoidal method.

### Homeostatic model assessment-insulin resistance (HOMA-IR)

Fasting serum insulin concentrations were measured in 20-week old TRIM31^−/−^ and WT mice by using an insulin ELISA kit (Abcam, Cambridge, UK). Insulin resistance was evaluated by the HOMA-IR score, calculated by fasting serum insulin (μU/ml)×fasting plasma glucose (mmol/l)/22.5.

### Biochemical analysis

Serum inflammatory cytokines, including IL-6 (interleukin-6), TNF-α (tumor necrosis factor α), IL-1β (interleukin-1β), and IL-10 (interleukin-10) were measured by the U-PLEX Assay Platform (Meso Scale Discovery, Rockville, MD), according to the manufacturer's instructions. All samples were tested in duplicate.

### Quantitative real-time RT-PCR

Total RNA was extracted from freshly isolated caecal samples by using TRIzol Reagent (Invitrogen, Carlsbad, CA). RNA was reverse transcribed from each sample using the Applied Biosystems cDNA Reverse Transcription kit (Applied Biosystems, Life Technologies). The cDNA was amplified with a SYBR® Green PCR Master Mix (RR420A, Takara Bio Inc., Otsu, Shiga, Japan). We used Oligo 7.0 software (Molecular Biology Insights, Inc., Cascade, USA) to design the sequences of the primers. The primer sequences for TNF-α, IL-1β, and β-actin genes for real-time RT-PCR are in Table S1.

### Western blot analysis

Protein extracts were separated by SDS-PAGE and transferred onto polyvinylidene fluoride membranes for incubation overnight at 4°C, with the corresponding primary antibodies for TNF-α (1:500; Abcam, Cambridge, UK), IL-1β (1:500; Abcam, Cambridge, UK), caspase-1 (1:1000; Abcam, Cambridge, UK), NLRP3 (1:1000; AdipoGen, CA), total IRS-1 (insulin receptor substrate-1) (1:1000; Abcam, Cambridge, UK), phosphorylated-IRS-1 (Ser307) (p- IRS-1) (1:200; Santa Cruz Biotechnology, Santa Cruz, CA, USA), total Akt and phosphorylated-Akt (Thr308) (p-AKT) (1:1000; Cell Signaling Technology, MA, USA), β-actin (1:1000; Abcam, Cambridge, UK) and tubulin (1:1000; ProteinTech, Wuhan, China), and appropriate secondary antibodies (1:5000; Abcam, Cambridge, UK) for 1-h at room temperature. Protein levels of TNF-α, IL-1β, caspase-1, NLRP3, IRS-1, and AKT were normalized to that of β-actin or tubulin.

### Gut microbiota analysis

#### PCR amplification and sequencing

Genomic DNA was extracted from caecal content of mice by using the EZNA DNA Kit (Omega Bio-tek, Norcross, GA), then the bacterial 16S ribosomal RNA (rRNA) gene targeting the V3-V4 region was amplified by using bar-coded universal primers, with 338F 5′-ACTCCTACGGGAGGCAGCA-3′ and 806R 5′-GGACTACHVGGGTWTCTAAT-3′. PCR reactions were performed in triplicate with the following mixture: 10 ng template DNA, 2 μl dNTPs (2.5 mmol/l), 0.8 μl forward primer (5 μmol/l) and 0.8 μl reverse primer (5 μmol/l), 0.4 μl FastPfu Polymerase, 4 μl 5 × FastPfu Buffer, and PCR-grade water in a final volume of 20 μl. Reactions were performed with the following cycling conditions: 95°C for 3 min, followed by 25 cycles at 95°C for 30 s, 57°C for 30 s, and 72°C for 45 s, with a final extension of 10 min at 72°C. Replicate amplicons were pooled and bead-purified by using the AxyPrep DNA Gel Extraction Kit (Axygen Biosciences, Union City, CA). Reaction products were pair-end sequenced by using Illumina MiSeq technology (Illumina Inc., San Diego, CA) (Caporaso et al., 2012).

#### Sequence analysis

The 16S rRNA gene raw reads were analyzed by using Quantitative Insights Into Microbial Ecology (QIIME, http://bio.cug.edu.cn/qiime/) (Caporaso et al., 2010). Operational taxonomic units (OTUs) were created by clustering the reads with 97% similarity by using UPARSE v7.1 (http://drive5.com/uparse/). The rarefaction analysis and Shannon diversity index were calculated with representative sequences of OTUs and their relative abundance determined by QIIME (Caporaso et al., 2010) RDP Classifier (http://rdp.cme.msu.edu/) was used to analyze the phylogenetic affiliation of each 16S-rRNA gene sequence, with confidence threshold of 70% against the silva (SSU115) 16S rRNA database (Amato et al., 2013). PCoA plots were generated according to the matrix of distance calculated by using the weighted UniFrac algorithm (Lozupone et al., 2006). The heatmap profile was generated by using R (http://www.r-project.org/) and bacterial taxa differences were elucidated by the linear discriminant analysis (LDA) effect size (LEfSe). The LEfSe algorithm was used to draw the cladogram, with the Huttenhower Galaxy web application (The Huttenhower Lab, Boston, MA; http://huttenhower.sph.harvard.edu/lefse/) (Segata et al., 2011).

### Statistical analysis

Data were expressed as mean ± standard deviation (S.D.). Non-parametric variables were mathematically transformed to improve symmetry. Unpaired *t*-test was used to study differences in continuous variables between groups. Mann-Whitney *U*-test was performed to examine differences in bacterial composition between TRIM31^−/−^ and WT mice. The correlations between microbial composition and metabolic and inflammatory parameters were performed using Spearman's analysis. The statistical significance was determined by SPSS 21.0 (SPSS Inc., Chicago, IL) and *P* < 0.05 was considered statistically significant.

## Results

### TRIM31^−/−^ mice exhibited glucose intolerance and insulin resistance

**Figure 1:**
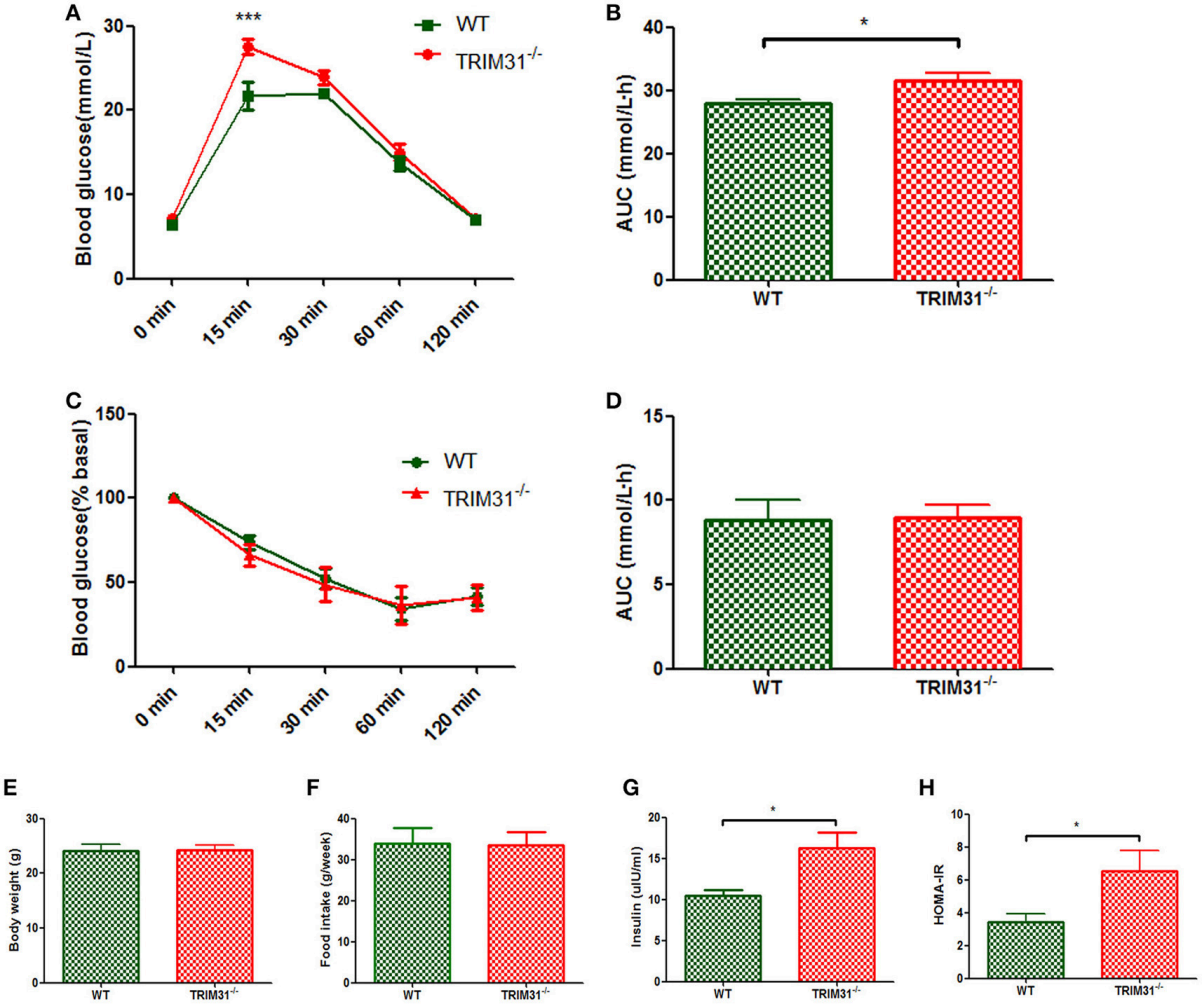
Glucose metabolism of TRIM31^−/−^ and WT mice at age 16 weeks. **(A)** Glucose tolerance test, **(B)** AUC for glucose tolerance, **(C)** Insulin tolerance test, **(D)** AUC for insulin tolerance test, **(E)** Body weight at age 16 weeks, **(F)** Food intake, **(G)** Serum insulin level, **(H)** HOMA-IR level. *n* = 10, in each group. ^*^P < 0.05 and ^***^P < 0.001 TRIM31^−/−^ vs. WT mice. AUC, area under the receiver operating characteristic curve; HOMA-IR, Homeostasis model assessment of insulin resistance; WT, wild-type; TRIM31, tripartite motif-containing protein 31.

There were no differences in body weight and food intake between TRIM31^−/−^ and WT mice from birth to the end of experimental period (Figures 1E,F). Glucose metabolism status was evaluated in TRIM31^−/−^ mice from 8 weeks old. However, no difference in glucose tolerance and insulin tolerance was observed between TRIM31^−/−^ and WT mice until 16 weeks old. For 16 week-old mice, blood glucose level was higher at 15 min after intraperitoneal glucose administration in TRIM31^−/−^ mice, compared with WT mice (*P* < 0.001; Figure 1A). Consistently, the AUC for IPGTT was greater in TRIM31^−/−^ mice (*P* < 0.05; Figure 1B). No difference in glucose levels of ITT was observed between TRIM31^−/−^ and WT mice (Figures 1C,D). Fasting serum insulin concentration and HOMA-IR were higher in TRIM31^−/−^ mice, indicating insulin resistance in TRIM31^−/−^ mice (*P* < 0.05, Figures 1G,H).

**Figure 2:**
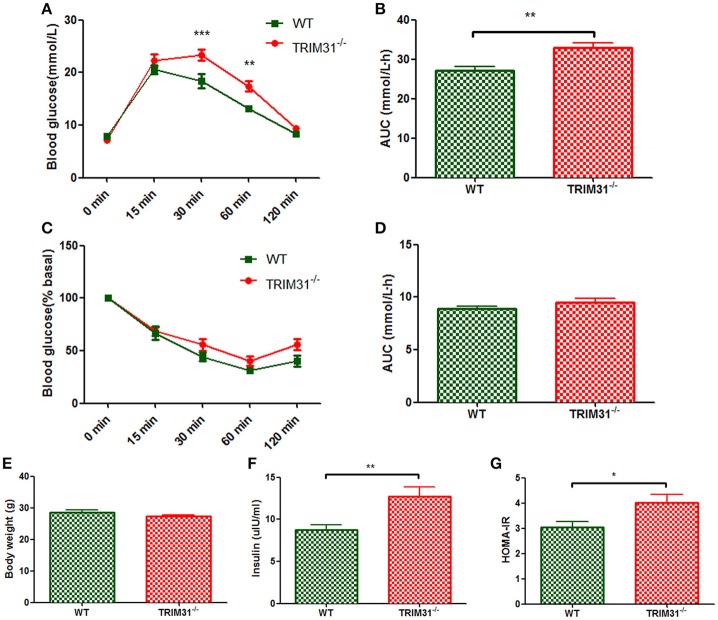
Metabolic parameters of TRIM31^−/−^ and WT mice at age 20 weeks. **(A)** Glucose tolerance test, **(B)** AUC for glucose tolerance, **(C)** Insulin tolerance test, **(D)** AUC for insulin tolerance test, **(E)** Body weight at age 20 weeks, **(F)** Serum insulin level, **(G)** HOMA-IR level. *n* = 10, in each group. ^*^P < 0.05, ^**^P < 0.01, ^***^P < 0.001 TRIM31^−/−^ vs. WT mice. AUC, area under the receiver operating characteristic curve; HOMA-IR, Homeostasis model assessment of insulin resistance; WT, wild-type; TRIM31, tripartite motif-containing protein 31.

For 20 week-old mice, there is no difference in body weight between TRIM31^−/−^ and WT mice (Figure 2E) the blood glucose value was higher at 30 min (*P* < 0.001) and 60 min (*P* < 0.01) after intraperitoneal glucose administration in TRIM31^−/−^ mice, indicating that impaired glucose tolerance was exacerbated in TRIM31^−/−^ mice with aging (Figure 2A). Consistently, the AUC for IPGTT was greater in TRIM31^−/−^ mice (*P* < 0.01; Figure 2B). However, no difference in blood glucose levels of ITT was found between TRIM31^−/−^ and WT mice (Figures 2C,D). Fasting serum insulin concentration (*P* < 0.01) and HOMA-IR (*P* < 0.05) were significant higher in TRIM31^−/−^ mice (Figures 2F,G).

### Characteristics of 16S rRNA gene sequencing

To profile gut microbiota structure differences between TRIM31^−/−^ and WT mice, bacterial 16S rRNA gene V3-V4 region was sequenced by Illumina MiSeq platform. A total of 515,839 high-quality sequences were obtained, with an average of 36,846 sequences per sample. The Good's coverage of each group was >97%, suggesting that the sequences identified represent most of the bacteria present in the samples. Taxonomy assignment showed the correlation between the duplicates to be >99.5% at any taxonomy level, indicating that the accuracy and reproducibility of sequencing was reliable for further analysis.

The OTU count was similar in TRIM31^−/−^ and WT mice (498.8 ± 67.6 vs. 511.9 ± 98.5). The diversity index (Shannon) and estimator of community richness (Chao) were uncomparable between the two groups, indicating the parallel community richness and diversity of gut microbiota between TRIM31^−/−^ and WT mice. Detailed information of these characteristics is shown in Table S2.

### Overall microbial structures of gut microbiota

The overall microbiota structure differed between TRIM31^−/−^ and WT mice at the phyla, family, and genus levels, respectively (Figure S2A–C). Principal coordinate analysis (PCoA) showed an overview of gut microbial dynamics associated with genotype. Bacterial structures differed between TRIM31^−/−^ and WT mice, as demonstrated by the first three principal component (PC) scores accounting for PC1 = 25.36%, PC2 = 18.16%, and PC2 = 12.84% of total variation, respectively. These findings indicate a statistically significant clustering by genotype (Figure S3).

### Phylotypes in TRIM31^−/−^ and WT mice

**Figure 3:**
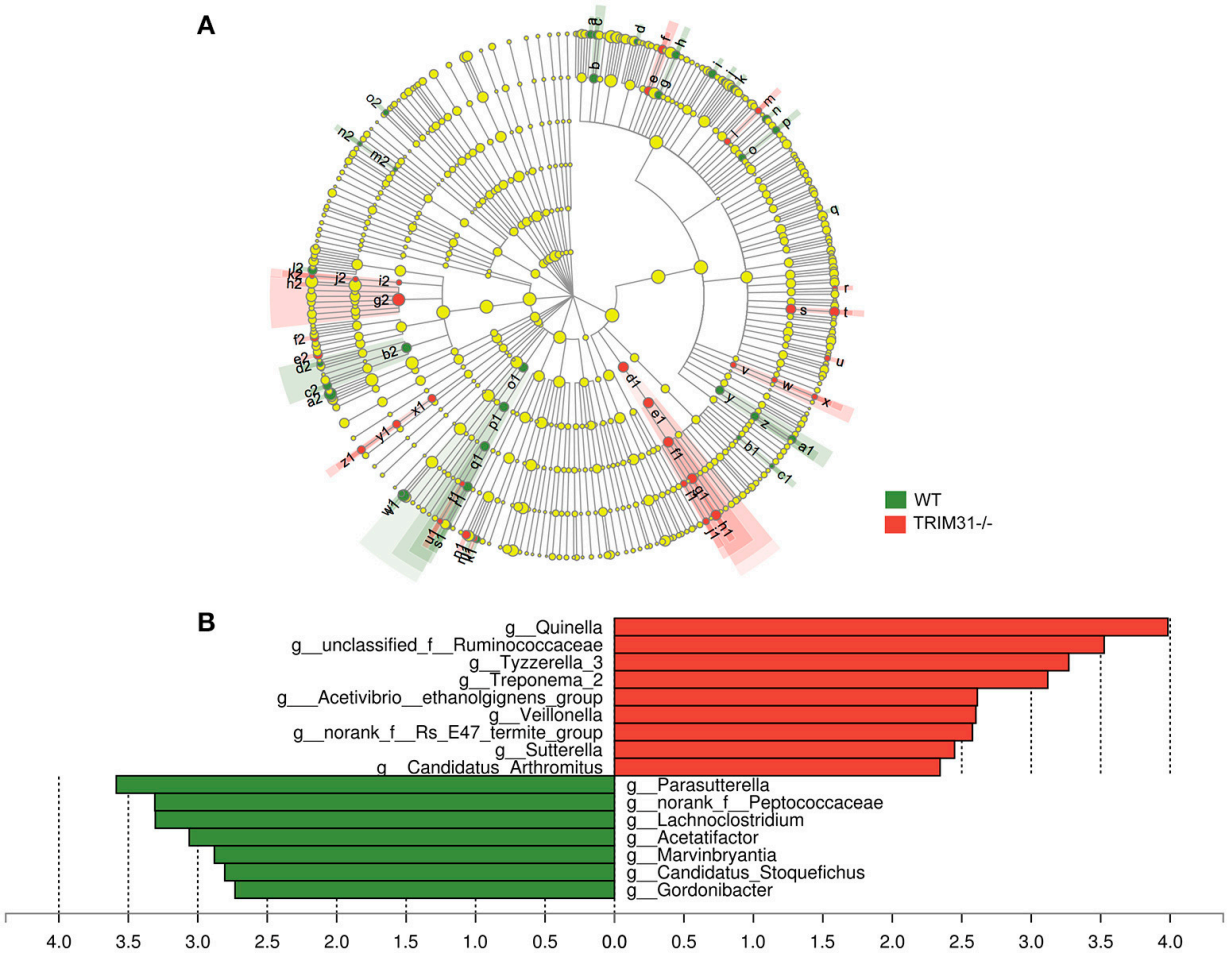
Different profiles of gut microbiota between TRIM31^−/−^ and WT mice. **(A)** Cladogram representation of gut microbiota taxa, from the phylum level to the bacteria level. Red indicates taxa enriched in TRIM31^−/−^ mice, and green indicates taxa enriched in WT mice. The diameter of each circle is proportional to the taxon's abundance. **(B)** Histogram of the LDA scores for differentially abundant taxa (red: TRIM31^−/−^ mice; green: WT mice). LDA scores were calculated by LDA effect size, by using linear discriminant analysis. *n* = 7, in each group. LDA, linear discriminant analysis; WT, wide-type.

**Figure 4:**
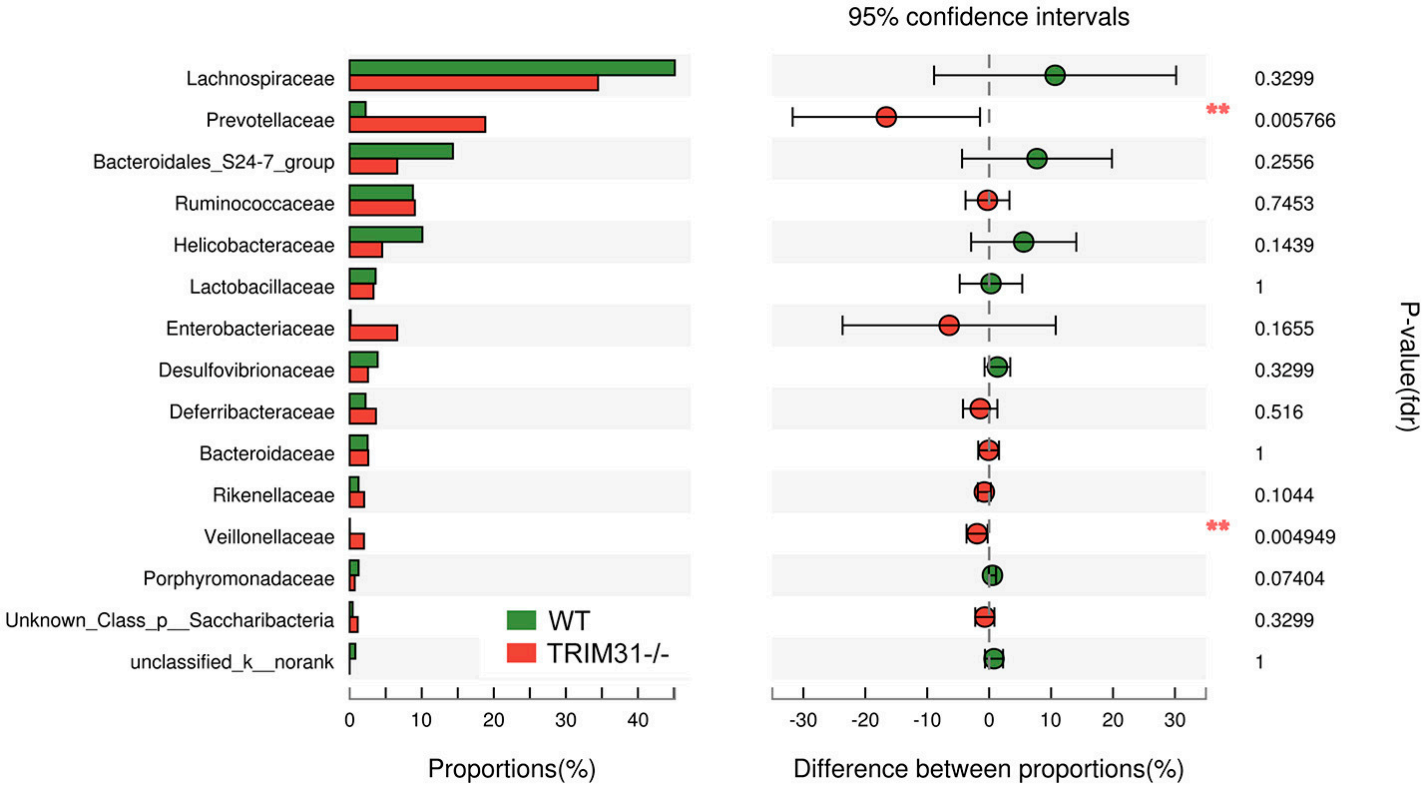
Significantly different phylotypes between TRIM31^−/−^ and WT mice at the family level. Data for mice are shown as relative abundance (%) of families in each group. *n* = 7, in each group. ^**^P < 0.01, TRIM31^−/−^ vs. WT mice by Mann-Whitney *U*-test.

A cladogram represents the significant structure of gut microbiota from the phylum level to the bacteria level (Figure 3A). The figure includes a list of the predominant bacteria in TRIM31^−/−^ and WT mice as determined by LEfSe. The greatest differences between TRIM31^−/−^ and WT mice at the family level are shown in Figure 3B. The differences in gut microbiota composition at the family level between TRIM31^−/−^ and WT mice are shown in Figure 4. It indicates significant variations in gut microbiota composition between the two groups. The proportions of Prevotellaceae (a family in the phylum Bacteroidetes) and Veillonellaceae (a family of Firmicute phylum) were both higher in TRIM31^−/−^ than WT mice.

### Inflammatory cytokines in TRIM31^−/−^ and WT mice

**Figure 5:**
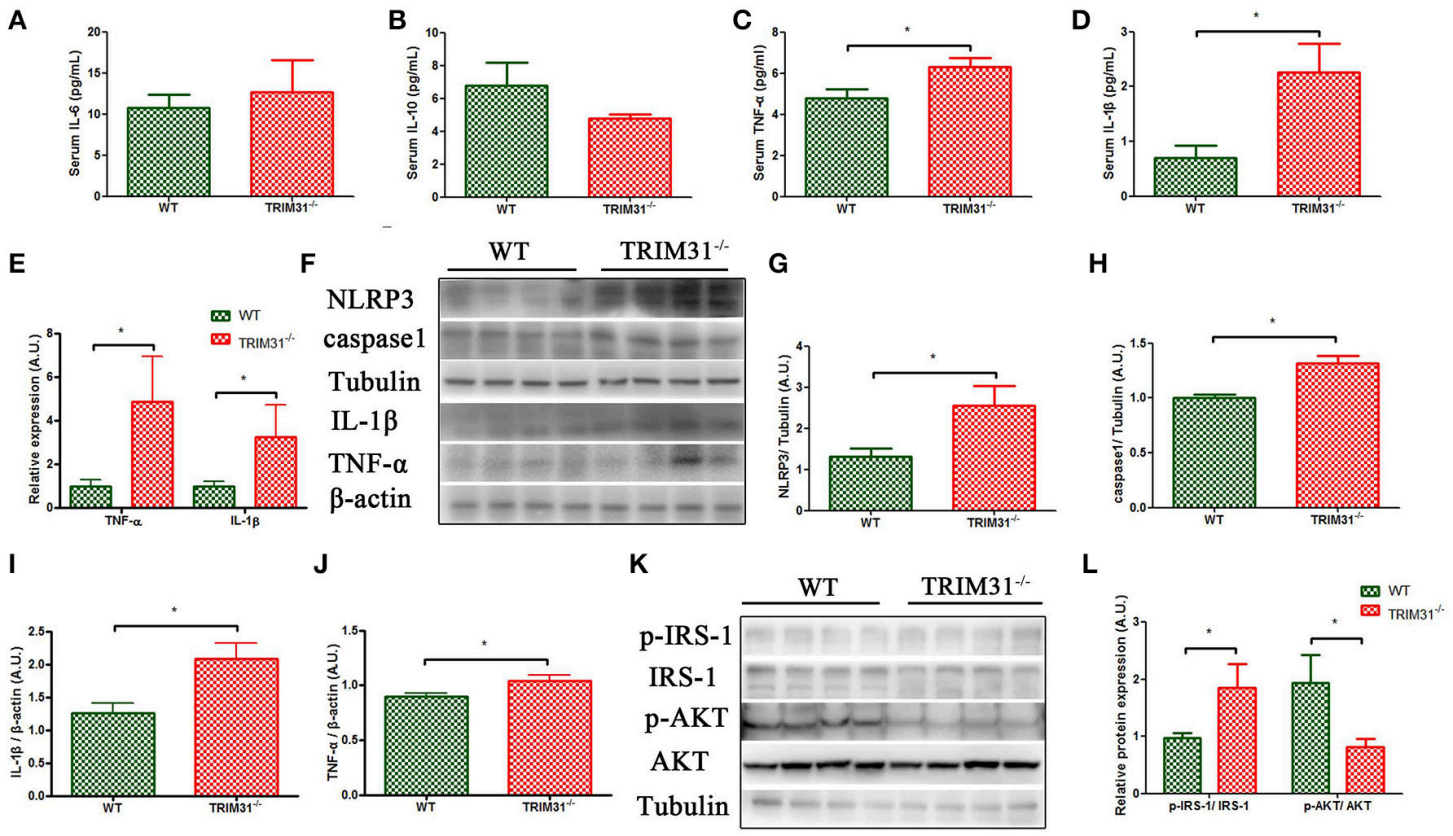
Elevated level of pro-inflammation cytokines in TRIM31^−/−^ mice. **(A–D)** Serum IL-6, IL-10, TNF-α, and IL-1β concentrations, **(E)** qRT-PCR of mRNA levels of TNF-α and IL-1β. **(F–J)** Western blot analysis of caecal protein levels of TNF-α, IL-1β, caspase-1,and NLRP3. **(K–L)** Western blot analysis of p-IRS-1 and p-Akt protein levels in visceral adipose tissue. *n* = 10, in each group. ^*^P < 0.05, TRIM31^−/−^ vs. WT mice. IL-6, interleukin-6; IL-10, interleukin-10; TNF-α, tumor necrosis factor-α; IL-1β, interleukin-1β; p-IRS-1, phosphorylated- insulin receptor substrate-1; p-Akt, phosphorylated-Akt.

We further measured serum inflammatory cytokines levels in 20-week old TRIM31^−/−^ and WT mice, including three pro-inflammatory cytokines (IL-6, TNF-α, and IL-1β) and one anti-inflammatory cytokine (IL-10). We found that serum IL-1β and TNF-α levels were higher in TRIM31^−/−^ mice, compared with WT mice. However, no statistical difference in IL-6 levels was observed between the groups. In addition, IL-10, as an anti-inflammatory cytokine, it showed a tendency to be lower in TRIM31^−/−^ mice (Figures 5A–D). Then, we found that TNF-α and IL-1β expressions were significantly higher in caecal samples from 20-week old TRIM31^−/−^ than WT mice (Figures 5E,F,I,J). NLRP3 inflammasome is a multi-protein platform which comprises NLRP3, ASC, and caspase-1. We detected the protein expression of NLRP3 and caspase-1 in caecal tissue of TRIM31^−/−^ and WT mice and found that NLRP3 and caspase-1 protein expressions were significantly upregulated in TRIM31^−/−^ mice, compared with WT mice, indicating that TRIM31 deficiency could lead to the activation of the inflammasome (Figures 5F–H).

### Decreased p-Akt/Akt protein levels in TRIM31^−/−^ mice

The insulin receptor signaling pathway is a major mechanism underlying the development of glucose intolerance and insulin resistance. The IRS-1 /phosphatidylinositol 3-kinase (PI3K)/Akt axis plays a key role in insulin receptor signaling transduction. Thus, we further examined the total and phosphorylation levels of IRS-1(Ser307) and Akt (Thr308) in visceral adipose tissue of TRIM31^−/−^ and WT mice. As shown in Figure 5, elevated p-IRS-1/IRS-1 protein expression, and decreased Akt Thr308 phosphorylation were found in TRIM31^−/−^ mice (Figures 5K,L).

### Correlation analysis between gut microbiota composition and inflammatory cytokines and insulin level

**Figure 6:**
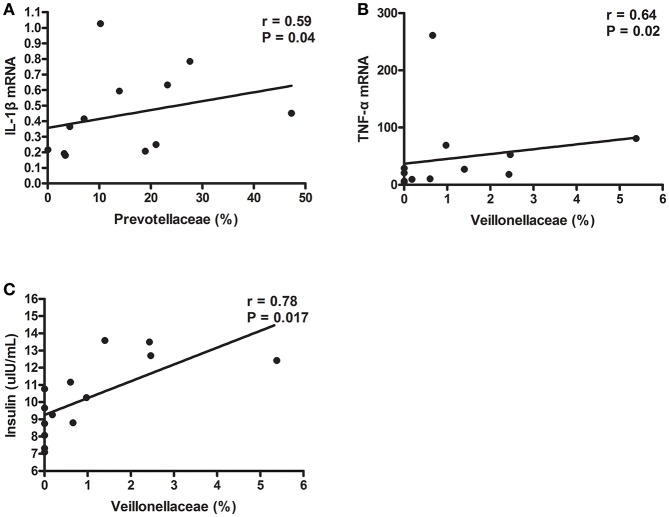
Gut microbiota taxa correlated with insulin and inflammation parameters in TRIM31^−/−^ mice. **(A)** Correlation of Prevotellaceae proportion with caecal IL-1β mRNA level. **(B)** Correlation of Veillonellaceae proportion with caecal TNF-α mRNA level. **(C)** Correlation of Veillonellaceae proportion with higher serum insulin level. Correlation analyses involved Spearman's correlation analyses. *n* = 7, in each group. TNF-α, tumor necrosis factor-α; IL-1β, interleukin-1β.

Then, we further investigated whether specific phylotypes, such as Prevotellaceae and Veillonellaceae, were associated with fasting serum insulin concentration and inflammatory cytokine levels. Strikingly, the proportion of Prevotellaceae, composed of four genera, Prevotella, Alloprevotella, Hallella, and Paraprevotella, was positively correlated with caecal IL-1β mRNA level (*r* = 0.59, *P* = 0.04) (Figure 6A). In addition, Veillonellaceae was associated with higher serum insulin concentration and caecal TNF-α mRNA expression (Figures 6B,C). These data showed that specific phylotypes were significantly correlated with serum insulin and inflammatory cytokine levels, and may play an important role in insulin resistance and activated inflammation status in TRIM31^−/−^ mice.

## Discussion

It is considered that environment, host genetics, and microbiota interact to maintain the homeostasis of gut, weight control, glucose tolerance, and insulin sensitivity (Spor et al., 2011). Modifications of these three components may trigger the development of obesity, insulin resistance, and diabetes mellitus. For the genetic factors, our study showed that TRIM31 knockout mice had glucose intolerance and insulin resistance. The metabolic disorders were characterized in 20-week-old TRIM31^−/−^ mice, with no difference in body weight between TRIM31^−/−^ and control animals. As demonstrated in other animal models (Barnard et al., 1993, 1998), insulin resistance precedes the development of obesity until age 20 weeks. In addition, to be consistent with previous studies, several knockout mice models also showed glucose intolerance and insulin resistance. In NLRP3 knockout mice, elimination of NLRP3 ameliorated obesity-induced inflammation and insulin resistance (Vandanmagsar et al., 2011), and caspase-1 knockout mice also improved glucose tolerance and insulin sensitivity than wild-type mice after feeding a high-fat diet (Stienstra et al., 2010). Cui et al. demonstrated that vitamin D3 1α-Hydroxylase knockout mice and Prion protein knockout mice developed insulin resistance (Cui et al., 2017; de Brito et al., 2017). Thus, impaired glucose metabolism has been observed and well-investigated in many transgenic animal models.

In recent decades, chronic inflammatory responses and oxidative stress are associated with the development of several metabolic disorders, such as obesity, insulin resistance, and T2DM. We found that TRIM31^−/−^ mice had impaired glucose tolerance and decreased insulin sensitivity, accompanied by moderate inflammation activation. Serum TNF-α and IL-1β concentrations and caecal TNF-α and IL-1β expressions were higher in TRIM31^−/−^ mice. The serum inflammatory cytokines levels were different from our previous work (Song et al., 2016), due to that the mice model was different. In our previous work, the female mice were mainly investigated and the age of the mice was 6 weeks old. More importantly, the previous study focused on an alum-induced peritonitis model, and found that TRIM31 could inhibit NLRP3 inflammasome activity in mouse peritonitis *in vivo*.

Many inflammatory cytokines have been reported to be associated with the development of metabolic disorders (Hotamisligil, 2006). Consistent with previous studies, some clinical trials suggest that increased TNF level results in impaired glucose homeostasis and insulin resistance in patients with T2DM (Gonzalez-Gay et al., 2010). IL-1β, as a prominent pro-inflammatory cytokine, can efficiently contribute to the generation of many inflammatory mediators (Arend et al., 2008). Our previous study demonstrated the potential functions of TRIM31 in the innate immune response, and TRIM31 deficiency facilitated NLRP3 inflammasome activation (Song et al., 2016). Consistent with previous study (Nie et al., 2016), elevated p-IRS-1/IRS-1 protein expression, and decreased Akt Thr308 phosphorylation were observed in TRIM31^−/−^ mice with impaired glucose tolerance and insulin resistance. Therefore, we speculated that TRIM31 deficiency could facilitate NLRP3 inflammasome activation and then have an important role in the development of metabolic disorders.

Gut microbiota consists of trillions of commensal micro-organisms residing within our intestines (Human Microbiome Project Consortium, 2012). All the genes of the gut microbiome represent at least 100-fold unique genes more than in the human genome (Qin et al., 2010). The gut microbiota, considered an “external” organ, participates in several aspects of host physiology and metabolism. Normal host functions depend on the overall balance in the composition of gut microbiota. Dysbiosis of gut microbiota have been found to contribute to an expanding list of chronic diseases including obesity (Seganfredo et al., 2017), diabetes mellitus (Qin et al., 2012), inflammatory bowel disease (Sartor, 2008), and systemic inflammatory response syndrome (Shimizu et al., 2006).

**Figure 7:**
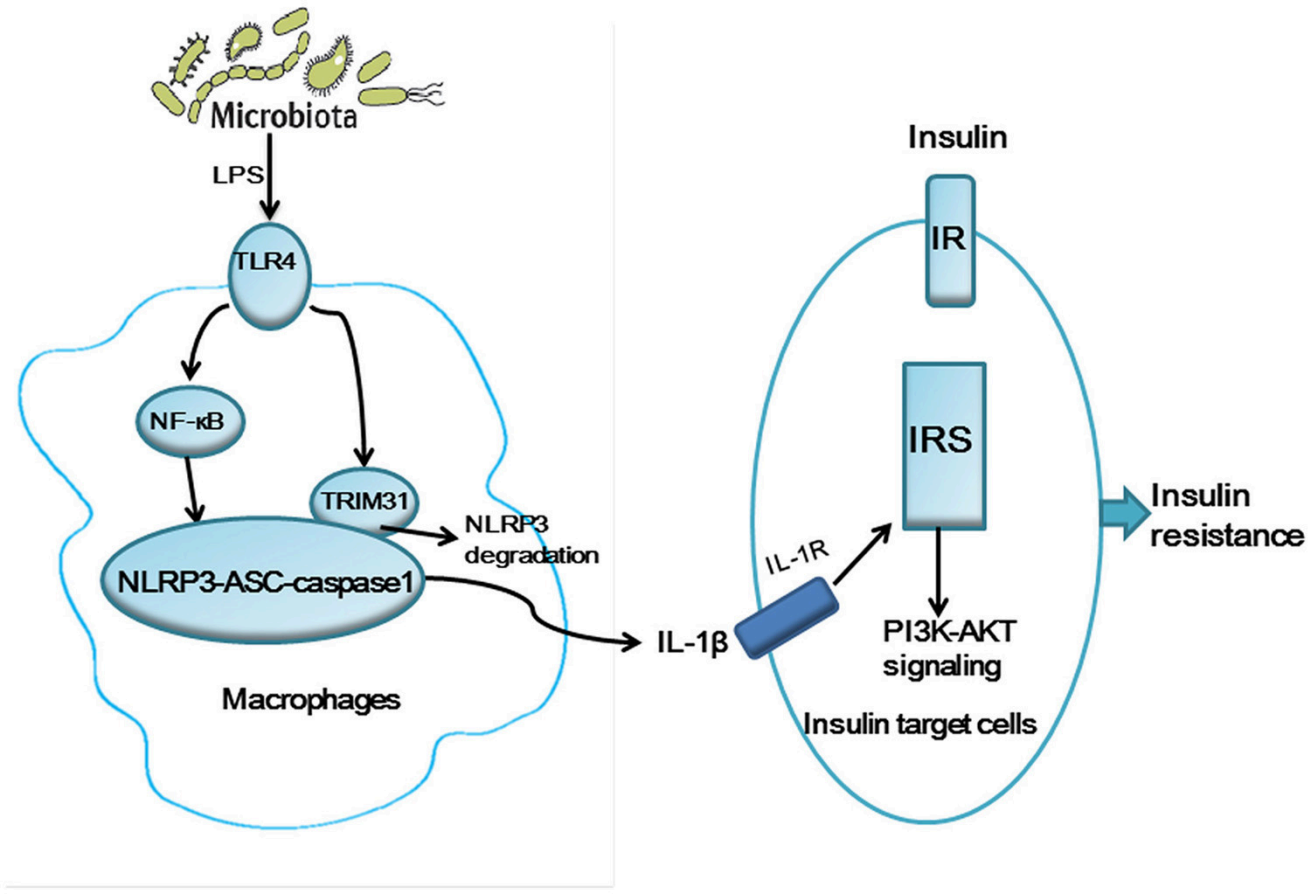
A hypothetical model for the pathogenesis of glucose intolerance and insulin resistance in TRIM31^−/−^ mice. Gut microbiota may generate LPS inducing NLRP3 inflammasome activation and the release IL-1β. TRIM31 binds to NLRP3 to promote proteasomal degradation of NLRP3. TRIM31 expression is markedly reduced in TRIM31^−/−^ mice, which promotes the release of IL-1β. The activated IL-1β induces insulin resistance by IRS1 and the PI(3)K–kinase Akt signaling pathway. LPS, lipopolysaccharide; IL-1β, interleukin-1β; IRS1, insulin receptor substrate 1.

Given the key role of the gut microbiota in the innate immune system and the potential functions of TRIM31 in the innate immune response, we aimed to profile gut microbiota in TRIM31^−/−^ mice. Consistent with previous studies, our study showed that the dominant phyla in the mice were Firmicutes, Bacteroidetes, and Proteobacteria (Karlsson et al., 2011; Evans et al., 2014). The relative abundance of Bacteroidetes was increased and that of Firmicutes and Proteobacteria decreased in TRIM31^−/−^ mice. To be consistent with previous study, Kellermayer et al. found a lower proportion of Firmicutes in Toll-like Receptor 2 (TLR2) deficiency mice, with increased proportion of Bacteroidetes (Kellermayer et al., 2011). It also showed that the proportion of Bacteroidetes was greater in TLR2^−/−^ mice (Caricilli et al., 2011). Bacteroidetes are gram-negative bacteria, with lipopolysaccharide (LPS) in the outer membrane. We also found increased relative abundance of Bacteroidetes in TRIM31^−/−^ mice and microbiota in TRIM31^−/−^ mice could produce more LPS. Activation of TLR4 by LPS promotes glycolysis, which contributes to nucleotide biosynthesis and enhanced ATP production, thus leading to NLRP3 inflammasome activation. Then, NLRP3 inflammasome activation can promote the release of IL-1β from macrophages, and activated IL-1β can activate c-Jun N-terminal kinase(JNK), IκB kinase (IKK), and the IRS-1 /PI3K/Akt axis via the IL-1 receptor (IL-1R). Engagement of the insulin receptor by IRS-1 is impaired with downregulation of the PI(3)K–kinase Akt signaling pathway (Wen et al., 2012). Thus, based on our findings and previous studies, a hypothetical model was tentatively proposed to illustrate the potential mechanism underlying how gut microbiota regulates glucose metabolism in TRIM31^−/−^ mice (Figure 7). This may be the underlying mechanism by which the TRIM31^−/−^ mice showed impaired glucose tolerance and insulin resistance.

## Conclusion

In conclusion, our study is novel in showing that TRIM31 deficiency is associated with impaired glucose metabolism and disrupted gut microbiota in mice, characterized by a clear difference in gut microbiota and inflammation activation, and gut microbiota is correlated with metabolic and inflammatory parameters. Our study provides a critical theoretical foundation for the putative roles of gut microbiota in the complicated molecular and cellular networks, which can contribute to building bridges between genotypes and phenotypes. A better understanding of the connections between TRIM31 deficiency and the development of impaired glucose metabolism and disrupted gut microbiota would be of great benefit and have potential implications for a wide range of common human metabolic disorders involving glucose intolerance, insulin resistance and diabetes mellitus.

## Author contributions

JC, FX, CC, MZ, LQ, JM, WS, XX, MZ (second author), and PH had substantial contributions to data curation, investigation, and methodology of the study. PH, JC, and MZ (second author) wrote, reviewed and edited the manuscript before submission. CG and YZ reviewed and edited the manuscript before submission. PH and JC had substantial contributions to conceptualization and formal analysis in the study. PH, MZ (second author), YZ, and CG made substantial contributions to supervision and validation. PH, CG, and YZ made substantial contributions to funding acquisition.

### Conflict of interest statement

The authors declare that the research was conducted in the absence of any commercial or financial relationships that could be construed as a potential conflict of interest.
