# Supplementary material for: TRIM31 Deficiency Is Associated with Impaired Glucose Metabolism and Disrupted Gut Microbiota in Mice
Source: Front Physiol. 2018 Feb 15;9:24. doi: 10.3389/fphys.2018.00024 (PMC5818424; doi:10.3389/fphys.2018.00024)
Supplement: Supplementary file 1 [file DataSheet1.DOC]

**Supplementary data**

**Figure Legends**

**Figure S1: Sequence analysis of WT mice and TRIM31-/- mice.** (A) The sequence peak of WT mice. (B) The sequence peak of TRIM31^-/-^ mice.

**Figure S2: The overall microbiota structure at different level.** (A) Overall structures of gut microbiota in each group at the phylum level. (B) Heatmap analyses of gut microbiota at the family level. The y axis is a neighbor-joining phylogenetic tree, each row is a different phylotype. The color of the spots in the right panel represents the mean relative abundance of the family in each group. (C) Community analysis pie-plot shows the overall microbiota structure at the genus level. n=7, in each group. WT, wide type; TRIM, TRIM31^-/-^ .

**Figure S3: Principal coordinate analysis plots in gut microbiota.** Weighted UniFrac PCoA plot based on OTU abundance. Each point represents the placenta microbiota of newborn mice, with TRIM31^-/-^ mice (red triangle) and WT mice (green circle). n=7 in each group. WT, wild-type.

**Table S1: Primer sequences for real-time RT-PCR.**

| **Genes** | **Forward primer** | **Reverse primer** |
| --- | --- | --- |
| TNF-α | CCCACGTCGTAGCAAACCA | ACAAGGTACAACCCATCGGC |
| IL-1β | GCAACTGTTCCTGAACTCAACT | ATCTTTTGGGGTCCGTCAACT |
| β-actin | TGTTACCAACTGGGACGACA | GGGGTGTTGAAGGTCTCAAA |

**Table S2: Sequencing data and comparison of estimator indices between TRIM31^-/-^ and WT mice.**

| **Estimators** | **WT** | **TRIM31^-/-^** | **P value*** |
| --- | --- | --- | --- |
| OTU | 511.88±98.48 | 498.84±67.65 | 0.778 |
| Ace | 412.10±71.85 | 410.99±31.18 | 0.972 |
| Chao | 415.25±71.31 | 418.64±34.43 | 0.914 |
| Coverage | 0.998±0.000 | 0.998±0.001 | 0.054 |
| Shannon | 4.36±0.41 | 3.89±0.44 | 0.084 |
| Simpson | 0.040±0.02 | 0.09±0.07 | 0.155 |

Data are mean ± SD, * Mann‐Whitney U test. The number of OTUs, richness estimator Chao, and diversity estimator Shannon were calculated at 3% distance. n=7 in each group. OTU, operational taxonomic unit.
