# Supplementary figures and images for: TRIM31 Deficiency Is Associated with Impaired Glucose Metabolism and Disrupted Gut Microbiota in Mice
Source: Front Physiol. 2018 Feb 15;9:24. doi: 10.3389/fphys.2018.00024 (PMC5818424; doi:10.3389/fphys.2018.00024)

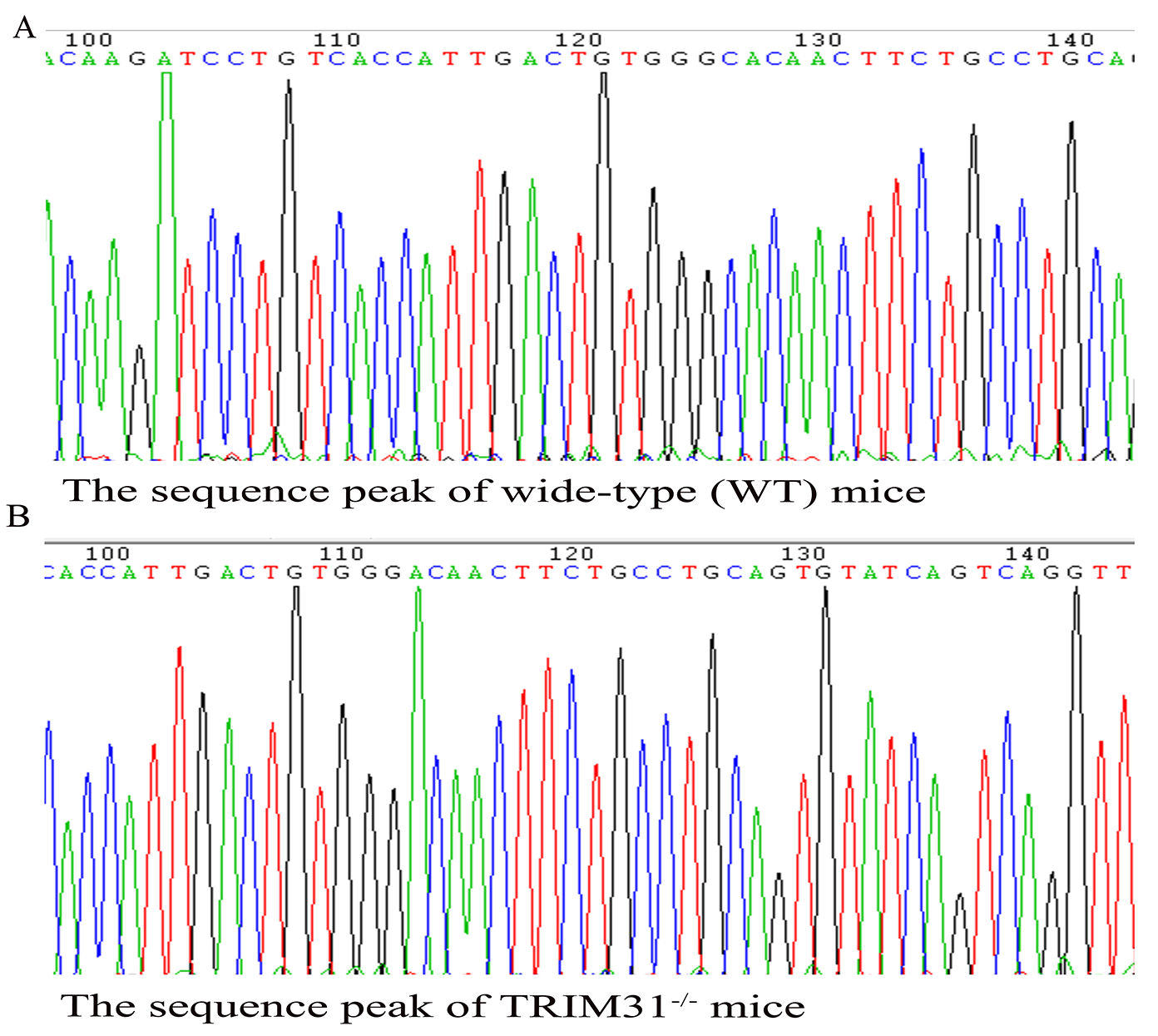

Supplement: Supplementary file 2 [file Image1.TIF]

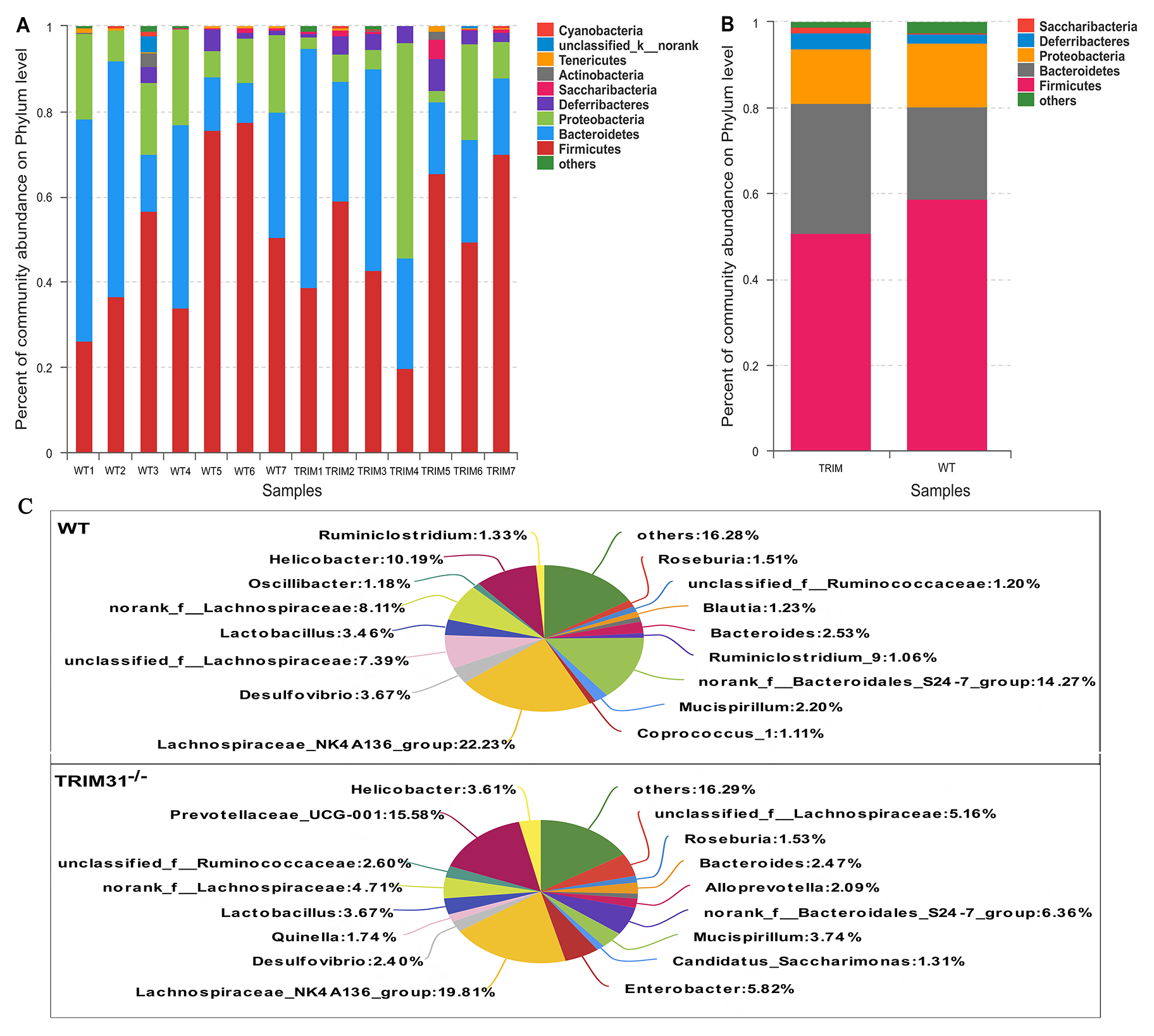

Supplement: Supplementary file 3 [file Image2.TIF]

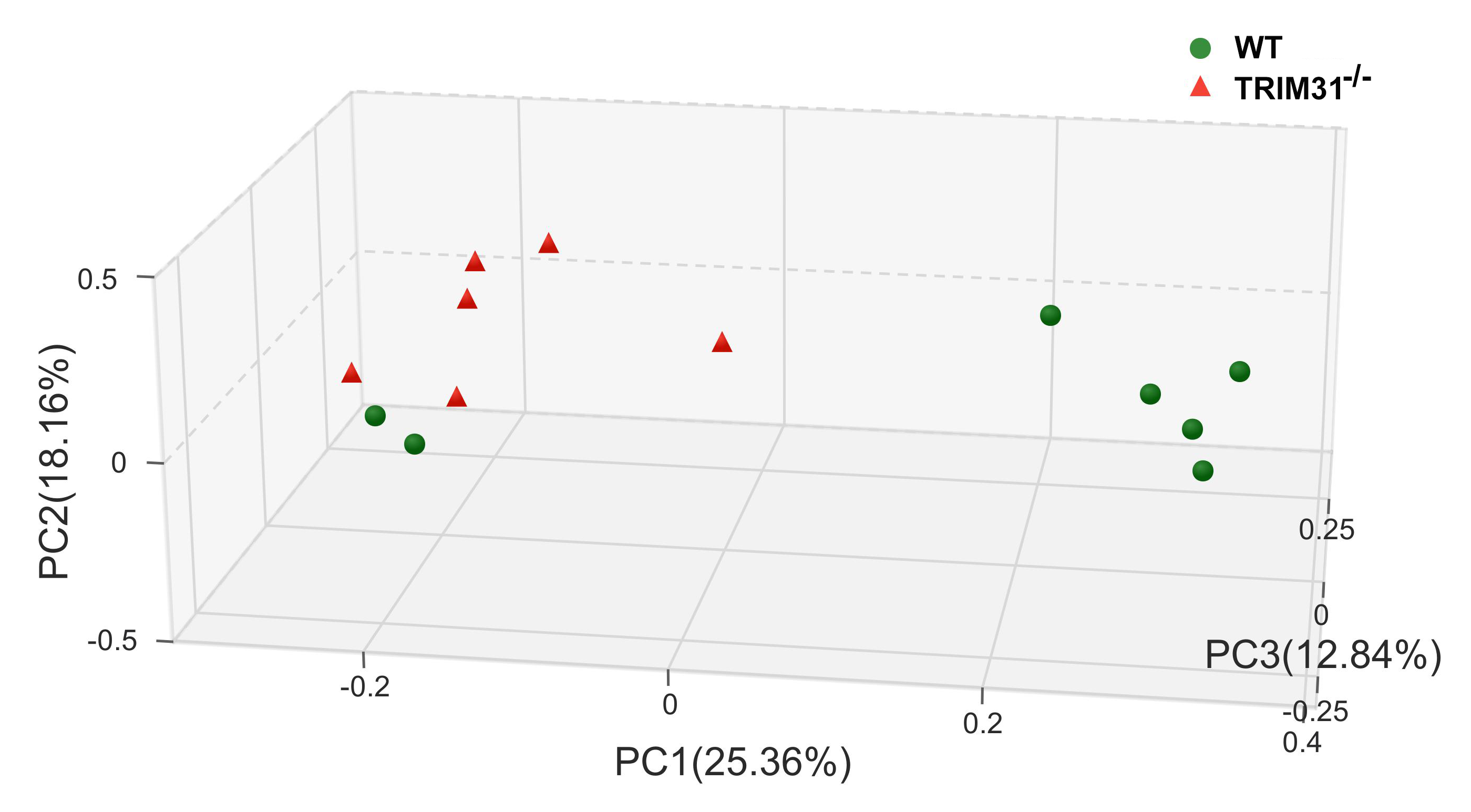

Supplement: Supplementary file 4 [file Image3.TIF]
